# Sustainable Electrochemical Depolymerization of Lignin in Reusable Ionic Liquids

**DOI:** 10.1038/s41598-017-05316-x

**Published:** 2017-07-11

**Authors:** Tobias K. F. Dier, Daniel Rauber, Dan Durneata, Rolf Hempelmann, Dietrich A. Volmer

**Affiliations:** 1 Institute of Bioanalytical Chemistry, Saarland University, Campus B2.2, 66123 Saarbrücken, Germany; 2 Institute of Physical Chemistry, Saarland University, Campus B2.2, 66123 Saarbrücken, Germany

## Abstract

Lignin’s aromatic building blocks provide a chemical resource that is, in theory, ideal for substitution of aromatic petrochemicals. Moreover, degradation and valorization of lignin has the potential to generate many high-value chemicals for technical applications. In this study, electrochemical degradation of alkali and Organosolv lignin was performed using the ionic liquids 1-ethyl-3-methylimidazolium trifluoromethanesulfonate and triethylammonium methanesulfonate. The extensive degradation of the investigated lignins with simultaneous almost full recovery of the electrolyte materials provided a sustainable alternative to more common lignin degradation processes. We demonstrate here that both the presence (and the absence) of water during electrolysis and proton transport reactions had significant impact on the degradation efficiency. Hydrogen peroxide radical formation promoted certain electrochemical mechanisms in electrolyte systems “contaminated” with water and increased yields of low molecular weight products significantly. The proposed mechanisms were tentatively confirmed by determining product distributions using a combination of liquid chromatography-mass spectrometry and gas-chromatography-mass spectrometry, allowing measurement of both polar v*ersus* non-polar as well as volatile *versus* non-volatile components in the mixtures.

## Introduction

The resources for global energy consumption and basic chemicals for industrial applications predominantly come from fossil fuels. More than 80% originate from crude oil, natural gas or coal^1^. Population growth and technical progress increase the demand for fossil fuels further and have led to increasing greenhouse gas (GHG) emissions. These anthropogenic GHG emissions possibly have irreversible effects, change the quality of life and the agricultural diversity, regionally and globally. Reduction of GHG emissions has been decided globally by political agreements^2^ and a successful achievement of emission targets is only possible through alternatives to fossil fuels including regenerative energy sources and sustainable processes to decrease the fossil fuel dependency of many industries. The biopolymer lignin with its polyaromatic structure^3, 4^, is a natural, sustainable precursor for fossil fuels^5^. The highly complex structure of lignin, which is embedded in the plant network^6^, complicates the simple conversion of the raw material to valuable chemicals. In the past, extraction processes, *e.g*. the Kraft process^7^ or other pulping techniques^8–11^, were mainly used in the paper and sugar cane milling industries to generate cellulose/sugar as target products. In these processes, lignin was predominantly incurred as waste, from which only a small part was processed in commercial applications (~1,000,000 tons/year; ≈2%)^12^, while the majority of the waste was simply combusted to produce energy. In the last two decades, intensive research on lignin conversion to valuable chemicals^13–19^ and biofuels^13, 20–23^ has been performed and the use of lignin as sustainable biopolymer has grown significantly. Among several possible technical processes, electrochemical conversion of lignin raw material has recently evolved as a potentially sustainable waste valorization procedure to generate valuable chemicals from its monomeric complement of components.

The electrochemical conversion rates and the range of generated products strongly depend on specific process parameters, particularly the nature of the electrolyte, the electrodes and the temperature. Water has been the most frequently used electrolyte for electrochemical lignin depolymerization. Schmitt *et al*. developed a system for selective synthesis of vanillin by using Ni and Co-based foams as electrodes and an anion exchange resin for adsorbing vanillin at considerable levels^24^. Other studies focused on untargeted conversions achieving polymer breakdown of more than 50% of the raw material^25–27^. Stiefel *et al*. were able to reduce the molecular weight of the initial lignin precursor by more than 93% in a short time period^27^. The cell voltage of the non-self-destructive water-based system remained at 1.23 V, however, thus restricting the variety of electrochemical reactions. Room temperature ionic liquids (RTIL) extended the electrochemical voltage window of the electrolyte^28, 29^, thus allowing further electrochemical reactions before the electrolyte started degrading. RTIL have been used for catalytic lignin depolymerisation^30–32^. In addition, the RTIL’s ability to dissolve significant amounts of raw lignin material^33–35^, its thermal stability^36^, promoting free radical reactions^37^ and its energy efficiency for biomass processing^38^ have promoted RTIL as promising electrolytes for lignin decomposition. Electrochemical decomposition using triethylammonium methanesulfonate as RTIL resulted in conversion rates of 3–6% (w/w) for low cell voltages (1–1.5 V)^39^, but increased to 20% (w/w) near the upper limit of the ionic liquid (1.7 V)^40^. In addition to common breakdown reactions (*e.g*., β-O-4 bond cleavages), previously unknown reductive reactions were observed; dehydroxylation, demethoxylation and hydrogenation were proposed as reductive mechanisms, after identifying the resulting degradation products^40^.

Untargeted lignin degradations result in highly complex product mixtures^41–43^, requiring analytical methods for characterization to be able to cover a broad range of chemical functionalities and molecular weights. Size exclusion chromatography (SEC) and Fourier-transform infrared spectroscopy (FTIR) have been applied to measure changes of molecular weight distributions^44–47^ and molecular vibrations^47–49^. These techniques, however, do not permit the determination of the individual components of the decomposition. Mass spectrometry (MS), in particular high resolution mass spectrometry (HRMS), and nuclear magnetic resonance (NMR) spectroscopy provide means of structure elucidation and compound assignment. These methods were used for deconvolution of complex lignin degradation mixtures^14, 18, 40, 50–53^ or determination of chemical modifications^14, 18, 19, 40, 52, 54, 55^. Using high resolution mass spectrometry at high mass accuracy and well-chosen atomic restraints permits the calculation of elemental formulae for the measured *m/z* features in the spectra. Importantly, structural isomers have been reported in the complex mixtures and most likely resulted from different degradation reactions^40^ and lignin structural diversities^41^. Therefore, separation techniques such as high-performance liquid chromatography (HPLC)^42, 43, 56^ or gas chromatography (GC)^57–59^ were required to separate structural isomers resulting from untargeted conversions. The ideal analytical methodology for successful characterization of lignin decomposition processes therefore comprises a conversion detection technique (GPC, FTIR), a technique for deducing structural information (MS, NMR) and a separation method to deconvolute the isobaric and isomeric content.

**Figure 1:**
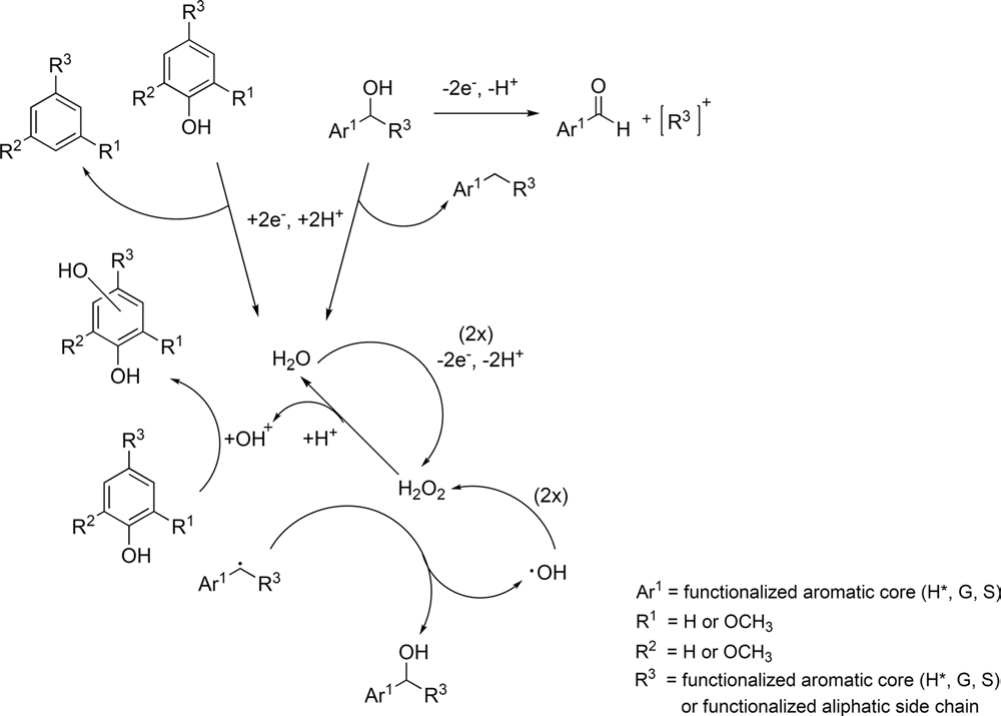
Abbreviated reaction scheme showing proposed electrochemical/radical mechanisms during lignin degradation. The numbers in parentheses give the equivalents of raw material needed for the reaction. Aromatic core units are defined as follows: (H*) 4-hydroxybenzyl, (**G**) 3-methoxy-4-hydroxybenzyl, (**S**) 3,5-dimethoxy-4-hydroxybenzyl.

In this study, we investigated the sustainability of the electrochemical conversion of commercial lignin using 1-ethyl-3-methylimidazolium trifluoromethanesulfonate ([emim][OTf]) and triethylammonium methanesulfonate (TMS) as electrolytes. The two IL, representing aprotic and protic solvent systems, respectively, were miscible with water, permitted the dissolution of large amounts of lignin, and allowed the application of high cell voltages. The electrochemically formed hydrogen peroxide (H_2_O_2_) was considered as one of the primary reaction partners for lignin degradation, allowing us to propose the degradation reactions summarized in Fig. 1. Of course, these proposed reactions likely only describe a subset of the full complement of available mechanisms.

We extracted the low molecular weight fraction from the residual polymer and recovered the ionic liquid by simple liquid-liquid extraction. The dissolved low molecular weight fractions were analyzed *via* LC-HRMS and GC-MS and the experimental results were used to tentatively confirm the proposed reactions in Fig. 1. The data also permitted the characterization of other reactions during the decomposition process. This study demonstrated that [emim][OTf] and TMS were fully functional as sustainable, reusable electrolytes and the new technique therefore permitted simple and flexible lignin decomposition in a non-toxic environment.

## Results and Discussion

### Characterization of the electrochemical process

**Figure 2:**
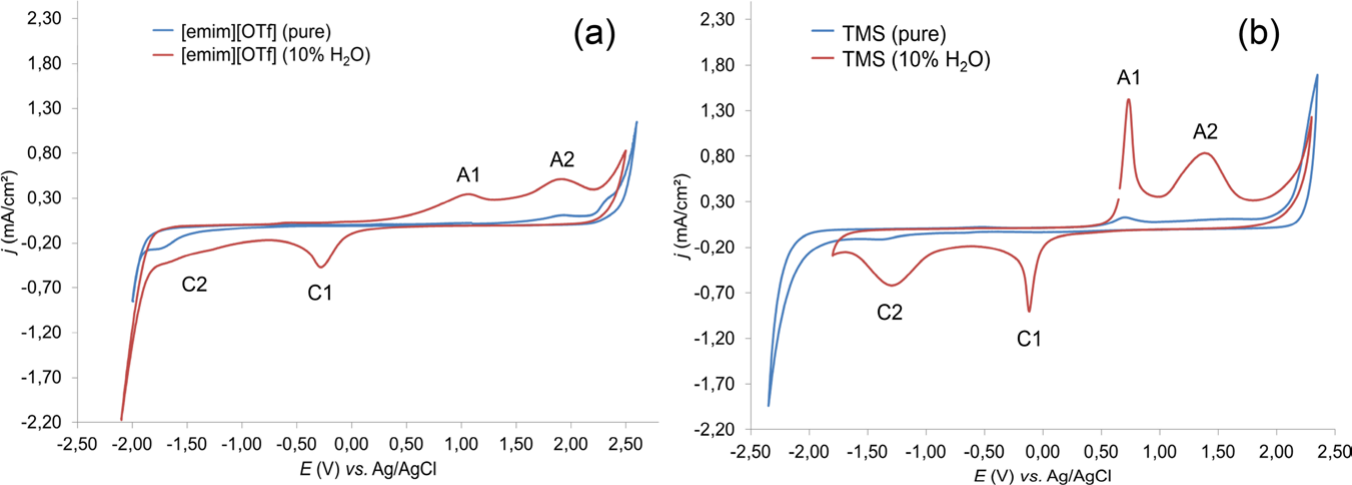
Cyclic voltammograms of pure (blue) and ‘water-contaminated’ (red) electrolytes (**a**) 1-ethyl-3-methylimidazolium trifluoromethanesulfonate ([emim][OTf]) and (**b**) triethylammonium methanesulfonate (TMS) (scan rates, 0.05 V/s).

The proposed mechanisms in Fig. 1 depend on the formation of H_2_O_2_. For detecting the electrochemically-formed H_2_O_2_, cyclic voltammetry (CV) of the two different electrolyte systems was implemented in this study (Fig. 2). Comparison of the pure and ‘water-contaminated’ [emim][OTf] electrolytes (Fig. 2(a)) exhibited two pairs of redox peaks for the ‘water-contaminated’ electrolyte. These two pairs were absent in the pure IL electrolyte system, indicating no significant electrochemical activity at the glassy carbon surface. Hence, they resulted from water addition to the system and were defined as subdivisions A1 and A2 for the anodic oxidation processes, and C1 and C2 for the cathodic reductions. The first oxidative process (A1) was attributed to formation of OH* radicals; by further increasing the potential, these radicals were oxidized to H_2_O_2_ (A2). The electrochemical formation of OH* and H_2_O_2_ from water in a two-step reaction has been previously shown^60^, which confirmed our assumption. At higher potentials (>2.3 V *vs*. Ag/AgCl), oxygen evolution was reached. On the other hand, the cathodic peak C1 at potential of −0.27 V *vs*. Ag/AgCl and C2 at −1.6 V *vs*. Ag/AgCl, were attributed to electrochemical reduction of the oxides to OH* and H_2_O_2_, respectively. The same behavior was seen for TMS (Fig. 2(b)). The pure TMS electrolyte system indicated no significant electrochemical activity, while addition of water triggered the electrochemical activity. Compared to [emim][OTf], the maximum current density of the TMS electrolyte for A1, A2 and C1 increased approximately 4, 1.6 and 2-fold, respectively. From the CV experiments, the oxidative transformation of water to H_2_O_2_ in the TMS system allowed us to propose the reactions in Scheme 1 for electrochemical lignin decomposition. Based on Mori *et al*. findings^61^, we determined the amount of electrochemically-produced H_2_O_2_ per hour by photometry measurements. After adding an acidified solution of titanium(IV) oxysulfate to the electrochemically-treated ‘water-contaminated’ TMS solution, the resulting yellow solution confirmed the electrochemical formation of H_2_O_2_ (resulting concentration after one hour: *c* = 0.13 μmol/ L; the calibration curve is shown in Figure S1).

In addition to the applied voltage, the amount of dissolved lignin is the main variable that controls the efficiency of the degradation process. The solubility of lignin in IL was significantly higher than in common preparations^62^. Thus, the improved solubility combined with the electrochemical stability (Fig. 2) presented a strong advantage of IL over solvents such as water. However, concentrated lignin solutions were highly viscous^63^ and therefore stirring was hampered in the electrolyte systems. The concentration of the working solutions was therefore restricted to 10% (w/w) and the process temperature was set to 65 °C. Under these mild conditions, the measured current was proportional to the extent of electrochemical conversion, as the surface of the electrodes remained the same. Table 1 summarizes the experimental parameters for each setup.

**Table 1:** Sample names and experimental parameters.

sample name	ionic liquid	applied voltage [V]	additive	temperature [°C]	duration [h]
[emim][OTf]-2.5	[emim][OTf]	2.5	—	65	24
[emim][OTf]-2.5-H_2_O	[emim][OTf]	2.5	H_2_O	65	24
TMS-2.5-H_2_O	TMS	2.5	H_2_O	65	24
TMS-2.5-H_2_O_2_	TMS	2.5	H_2_O_2_	65	24
TMS-0-H_2_O_2_	TMS	0	H_2_O_2_	65	24

**Figure 3:**
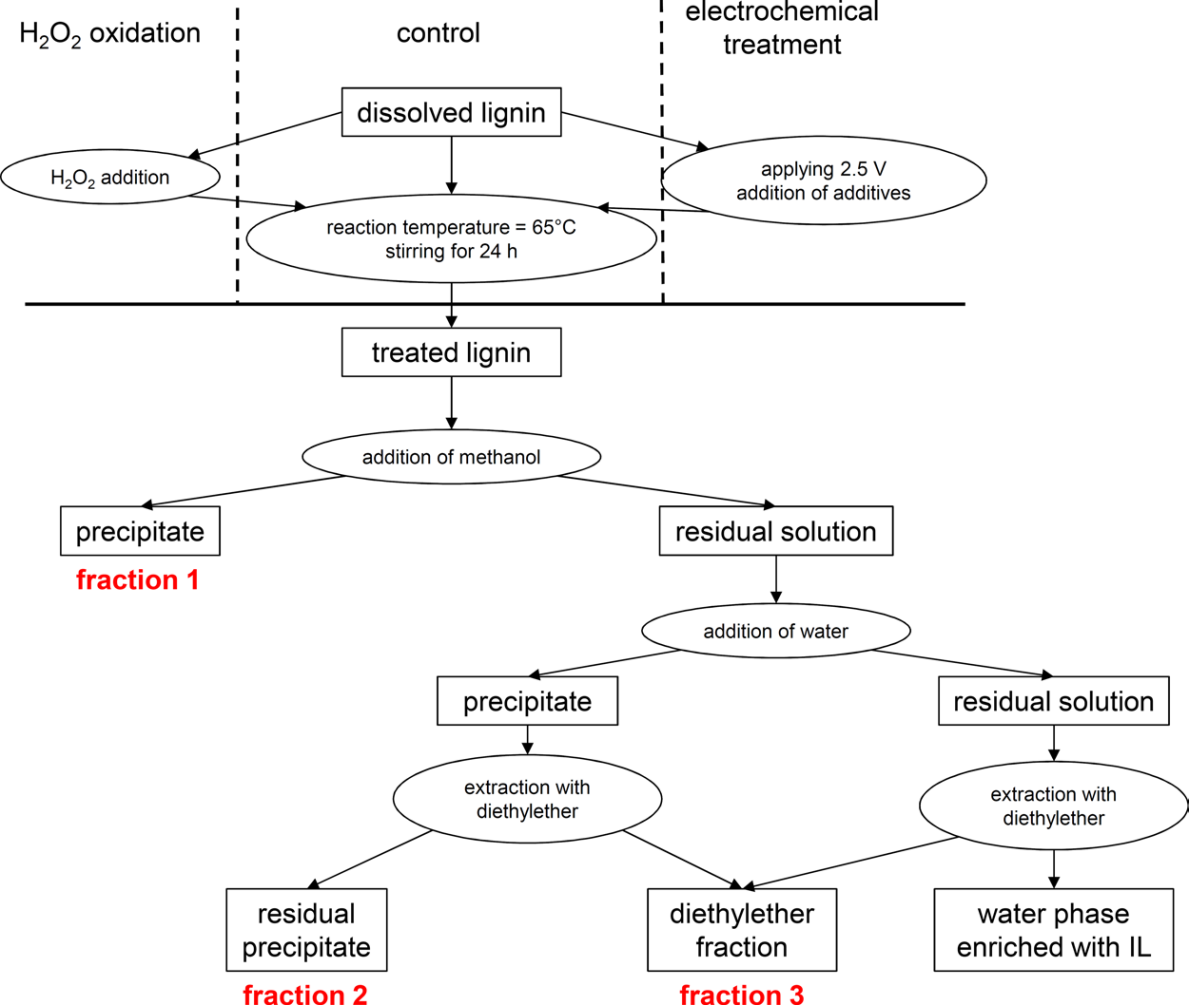
Schematic representation of the electrochemical degradation process including extraction steps.

The resulting mixtures were compared to a control sample, consisting of an electrochemically untreated lignin solution (UT), which was identical with respect to dissolution, temperature and liquid-liquid extraction steps. The molecular weight distributions of the precursor lignins were determined by gel permeation experiments, as follows: alkali lignin, *M*_W_ = 7435 g/mol, polydispersity index (PDI) = 3.98; organosolv lignin, *M*_W_ = 976 g/mol, PDI = 2.52. A flowchart of the process is illustrated in Fig. 3.

Diethylether was chosen as extraction solvent, since [emim][OTf] is miscible with common extraction solvents such as ethyl acetate^64^. NMR analysis of the recovered IL demonstrated the complete separation of degradation products and electrolytes, allowing reuse of the electrolytes in subsequent degradation reactions without purification (the NMR spectra are shown in Figures S2 and S3). The chronoamperograms of the different electrolyte systems clearly indicated the important role of water during the electrochemical decomposition process (data not shown). The electrochemical formation of H_2_O_2_ was consistent with previous results and has already been reported in the literature^65^. However, differences in current densities were observed. The protic behavior – or rather the H^+^ transport through the electrolyte – probably enhanced the electrochemical oxidation of lignin *via* smooth transition of protons formed during the degradation processes^66^ into the electrolyte system, and lignin decomposition caused by electrophilic substitution of the preferentially formed OH^+^ ions. The formation and reaction of these OH^+^ ions with lignin model compounds in acidic solvents was previously reported by Sun *et al*.^67^, supporting our assumptions for the ‘water-contaminated’ protic ionic liquids. However, the information from the chronoamperograms only provided information on the bulk electrochemical conversion. For a more detailed view of the lignin decomposition process and its underlying reaction mechanisms, other analytical techniques were necessary. Elemental analyses of the solid materials for each electrolyte system after liquid-liquid extraction showed increased oxygen content in comparison to control (Table 2). This increase for TMS-2.5-H_2_O was consistent with the reaction mechanisms described in Fig. 1. However, the increased nitrogen content for [emim][OTf] pointed to a possible interaction between IL and lignin, leading to chemical modification and degradation of the lignin unit. Therefore, secondary reactions during lignin degradation were present in aprotic IL electrolyte systems. We suggest that the imidazolium ring undergoes a ring-opening mechanism, either caused by radical lignin species or other unknown electrochemical water transformations. Czerwicka *et al*. observed a similar behavior for imidazolium based ionic liquids during oxidative degradation processes^68^. For TMS-2.5-H_2_O, the protic IL did not undergo any secondary reactions with the dissolved lignin, as the measured nitrogen content was comparable or even lower than in the untreated control fractions. However, the measured nitrogen content also increased for the TMS system after adding significant amounts of H_2_O_2_ to the reaction (*c* = 0.88 mol/L). This large amount of H_2_O_2_ was able to promote secondary reactions of the ionic liquid with the *in situ* formed degraded lignin species. Similar results were found for a currentless H_2_O_2_ approach. It was therefore concluded that the mechanistic role of H_2_O_2_ was very different for electrochemically-generated or directly added H_2_O_2_ (at comparable reaction conditions). Electrochemically-generated H_2_O_2_ from water predominantly underwent the reaction mechanisms described in Fig. 1, while directly added H_2_O_2_ exhibited additional oxidative mechanisms, thereby partially consuming the ionic liquid.

**Table 2:** Elemental analyses, yields (Y) and IL recovery rates (rec IL) for each electrolyte system: UT, lignin control; [emim][OTf]-2.5; [emim][OTf]-2.5-H_2_O; TMS-2.5-H_2_O; TMS-0-H_2_O_2_; TMS-2.5-H_2_O_2_.

		UT			[emim][OTf]-2.5			[emim][OTf]-2.5-H_2_O			TMS-2.5-H_2_O			TMS-0-H_2_O_2_			TMS-2.5-H_2_O_2_		
Fraction		1	2	3	1	2	3	1	2	3	1	2	3	1	2	3	1	2	3
C	wt.	58.42 ± 0.20	63.99 ± 0.46	64.96 ± 1.22	55.33 ± 0.97	54.45 ± 2.52	57.29 ± 0.75	56.65 ± 0.27	62.67 ± 0.31	54.37 ± 0.82	58.4 ± 0.96	63.35 ± 0.09	62.39 ± 2.9	57.47 ± 4.1	59,88 ± 6.23	56,91 ± 8.31	50.41 ± 1.01	57.72 ± 1.46	58.99 ± 4.81
H		5.01 ± 0.06	5.62 ± 0.01	6.96 ± 0.05	6.09 ± 0.17	5.75 ± 0.82	6.14 ± 0.13	5.25 ± 0.18	5.77 ± 0.07	5.22 ± 0.27	5.68 ± 0.11	5.93 ± 0.11	6.19 ± 0.73	7.5 ± 0.26	5,90 ± 0.09	7,89 ± 0.14	6.16 ± 0.2	6.3 ± 0.7	6.71 ± 0.11
N		0.51 ± 0.12	0.4 ± 0.37	0.32 ± 0.04	1.46 ± 0.43	2.49 ± 1.09	2.1 ± 0.43	1.1 ± 0.09	0.88 ± 0.02	1.71 ± 1.37	0.28 ± 0.05	0.5 ± 0.02	0.22 ± 0.09	0.49 ± 0.77	1,67 ± 1.07	2,07 ± 1.38	0.64 ± 0.08	1.85 ± 0.74	1.26 ± 0.56
O^[a]^		36.06 ± 0.38	29.99 ± 0.84	27.76 ± 1.31	37.12 ± 1.57	37.31 ± 4.43	34.47 ± 1.31	37 ± 0.54	30.68 ± 0.4	38.7 ± 2.46	35.64 ± 1.12	30.22 ± 0.22	31.2 ± 3.72	34.54 ± 5.13	32.55 ± 7.39	33,13 ± 9.83	42.79 ± 1.29	34.15 ± 2.9	33.04 ± 5.48
Y	[mg]	173 ± 5	629 ± 3.5	163 ± 15	610 ± 27	320 ± 13	17 ± 2	241 ± 23	495 ± 11	200 ± 16	145 ± 16	593 ± 8	199 ± 24	102 ± 11	518 ± 24	180 ± 6	2.5 ± 2	628 ± 13	119 ± 10
Total		965 ± 23.5			947 ± 42			936 ± 50			937 ± 48			800 ± 41			749 ± 25		
rec IL	[%]	99.2 ± 0.4			98.2 ± 0.2			98.6 ± 0.1			99.3 ± 0.2			97.9 ± 0.7			98.3 ± 0.6		

Experiments were performed in duplicate.

[a]: oxygen content was calculated as the residual w eight percentage.

Nevertheless, the important role of water for the electrolyte system was evident during electrolysis in the aprotic electrolyte system, where the water-free system mainly resulted in higher yields of fraction 1. Very similar behavior was observed by di Marino *et al*. for electrochemical degradation of lignin in deep eutectic solvents, which were spiked with water^69^. Higher yields of methanol and diethylether-soluble products were desired for the lignin degradation, however, as breakdown products of lower molecular weight are more soluble in these common solvents than the lignin oligomers and polymers. The outcome of the water-free system indicated a chemical modification of lignin and subsequent repolymerization reactions instead of breakdown reactions. Single electron transfers (SET)^39, 40^ under the experimental conditions will generate radical lignin species that have the potential to undergo radical intra- and intermolecular reactions as well as radical reactions with the IL (Table 2, indicated by increasing nitrogen content). The *in situ* electrochemically formed species (Fig. 2) trapped the free radicals, however, resulting in formation of oxidized lignins. These scavenging mechanisms and hydrogen peroxide oxidations were consistent with the observed increased oxygen content for each of the investigated electrolyte systems. Importantly, the proposed reactions only describe a small proportion of the full complement of available mechanism. For example, the dehydroxylation mechanism is not restricted to hydroxylated aromatic cores. Dehydroxylations in side chains or demethoxylations are equally possible electrochemical reductions. As a result, the “water-contaminated” IL electrolyte system was able to dissolve significantly larger amounts of lignin than common electrolyte systems, and it was also able to degrade lignin to a significant extent. Furthermore, TMS was more suitable as electrolyte system. The presence of degradation products resulting from secondary reactions with the electrolyte can be excluded for TMS-2.5-H_2_O and they were virtually fully recovered after liquid-liquid extraction. Using H_2_O_2_ directly as reactive species significantly reduced the amount of recovered IL. Similar to [emim][OTf] systems, TMS was able to interact with certain lignin species and undesirable, H_2_O_2_-promoted side reactions occurred.

The discrepancy seen for C, H, N and O content as well as yield for the untreated lignin sample (Table 2) can be readily explained by inhomogeneities of the lignin sample, which is consistent with the literature^70, 71^. These inhomogeneities also impacted the electrochemical degradation process and complicated the interpretation of the results of the electrochemical decomposition process and the analysis of the low molecular weight fractions. The general efficiency of the implemented electrolyte systems for electrochemical lignin degradation can be described either by the yield of fraction 1 or *via* the yield of fraction 3. Repolymerization reactions of oligomers, however, complicate this interpretation of the process. Fraction 2 mainly consisted of higher mass oligomers, since methanol exhibited moderate solubility of the formed solids in each of the investigated electrolyte system. Fraction 2 can therefore be described as a “transitional phase”, where oligomers are either transformed to higher mass polymers, or where the oligomers are degraded to low molecular weight products. As a result, the yields seen for fraction 3 were used as a rough estimate of the electrolyte system’s ability for lignin degradation. TMS-2.5-H_2_O and [emim][OTf]-2.5-H_2_O exhibited electrochemical lignin decomposition rates of 22 and 23% (w/w), respectively, while [emim][OTf]-2.5 resulted in a decrease of low molecular weight products of 90% (w/w). TMS-2.5-H_2_O_2_ also decreased the yield of fraction 3 by 10%. In addition, the total yields for TMS-H_2_O_2_ systems did not reach the expected amounts (product recovery >85%). Therefore, full degradation of certain lignin units to highly volatile or gaseous compounds had to be taken into account for the TMS-H_2_O_2_ systems. A more detailed analysis of the efficiency and selectivity of the process was possible from detailed analysis of the fractions containing low molecular weight products, by using LC-MS, as described in the following section.

### Distribution of polar, non-volatile products of low molecular weight

**Figure 4:**
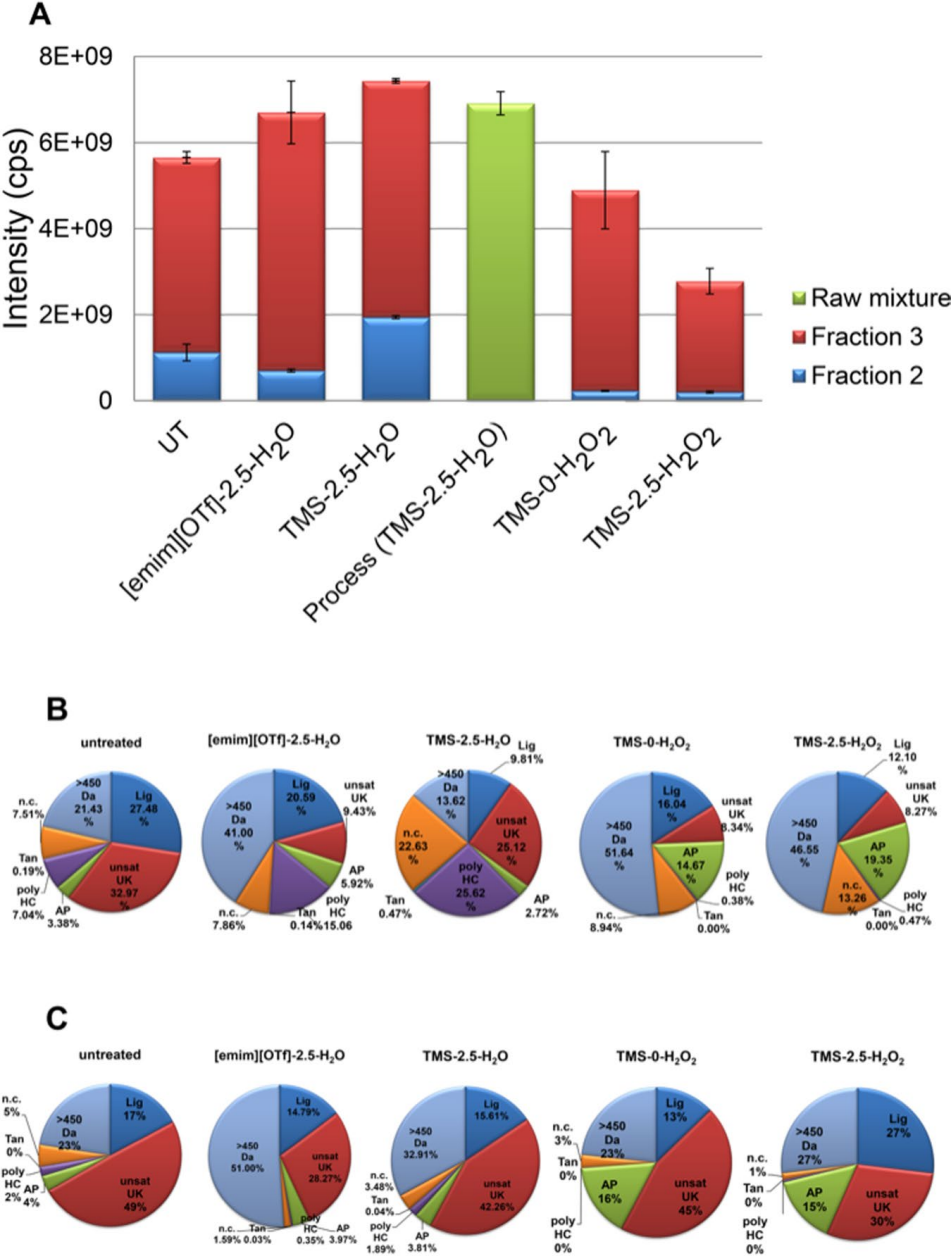
(**A**) Total intensities for relevant lignin degradation products. (mass concentration, *β* = 100 μg/ mL) (**B**) Percent distributions of chemical classes for fraction 2. (**C**) Percent distributions of classes for fraction 3. Distributions were restricted to *m/z* ≤ 450, unless otherwise specified. Alkali lignin was used for all experiments.

The conversion of lignin to small molecules (<1000 Da) was the primary aim of our study. The analysis of this low molecular weight fraction was conducted using LC-MS for the polar and moderately volatile/in-volatile compounds fraction. Atmospheric pressure chemical ionization (APCI) was chosen as ionization technique for LC-MS, as multiply-charged ions for higher molecular weight oligomers are excluded with this technique and thus all measured *m/z* features could be related to small lignin-related molecules. Lignin’s large diversity of chemical functionalities and the possibilities for formation of a significant number of isobaric compounds required gradient elution in combination with a stationary phase of appropriate selectivity. Chromatographic columns with large variety of interaction mechanisms (mixed-mode columns)^72–74^ enabled deconvolution of complex lignin degradation mixtures and were used in this study. The IL columns required frequent regeneration of the stationary phase after intensive usage, as ion-exchange was one of the occurring interaction mechanism^73^. This was readily achieved by injection of the raw mixture solution. A further degree of information and interpretation of the reaction mechanisms was obtained by combining the selective IL chromatographic separations with enhanced mass defect filtering^40^ as well as van Krevelen plots^75–78^. This combination allowed classification of the measured *m/z* features into specific compound classes. The combined use of O/C *versus* H/C and KMD[CH_2_] *versus* KMD[C_7_H_7_O_2_] diagrams uncovered the formation of products of the following distinct substance classes: lignins (Lig), unsaturated unknowns (unsat UK), alkylphenolics (AP), saturated unknowns (sat UK), cycloalkanes (CA), polycyclic hydrocarbons (poly HC), polysaccharides (PolyS), tannins (Tan), and non-classifiable compounds (n.c.) (Fig. 4). The extracted mass spectra from each chromatogram allowed the calculation of relative sums for compound classes and thus a comparison of the effectiveness of the different electrochemical systems (chromatograms and mass spectra are shown in Figure S4).The measured distributions were also used to reveal specific chemical transformations of lignin during the electrochemical treatments. Higher mass oligomers were primary detected for [emim][OTf]-2.5-H_2_O, indicating either slower lignin degradation as compared to TMS-2.5-H_2_O, or the presence of repolymerization reactions of radical low molecular weight lignin species. In both cases the lack of system-wide H^+^ transport – as required for most of the electrochemical degradation reactions – was seen as the primary reason for these phenomena. For TMS-H_2_O_2_ systems, higher mass oligomers were primary detected in fraction 2. This increased abundance resulted from either improved degradation of fraction 1 or repolymerization reactions of low molecular weight lignin species. The measured distributions also permitted a mechanistic rationale for the observed strong abundances of somewhat unexpected chemical classes. For example, the large abundance of polycyclic hydrocarbons in fraction 2 for [emim][OTf]-2.5-H_2_O and TMS-2.5-H_2_O can be readily explained by either electrochemically formed reactive species or by the reductive pathways in Fig. 1, respectively. Here, polycyclic hydrocarbons were defined as features in the mass spectra with average H/C ratios of ~1 and low O/C ratios of up to 0.15. These are only possible *via* dehydroxylation or demethoxylation reactions. However, the lignin structure primary consists of phenolic components, interlinked by functionalized aliphatic chains^4^, which somewhat contradicts the formation of abundant polycyclic hydrocarbons during electrochemical treatment. Therefore, electrochemical reactions of mono-aromatic molecules to polycyclic compounds should be considered as a source of these compounds. The exact formation mechanism and required functional groups for cyclization, however, remain unknown and require further study. The measured distributions for TMS-H_2_O_2_ systems showed a significant increase of the AP fraction (see Fig. 4C). This increase indicated hydrogen peroxide’s ability to promote further dehydroxylations and demethoxylations. On the other hand, TMS-2.5-H_2_O_2_ was also able to increase the relative abundance of the Lig compound class. This increase showed that electrochemically formed H_2_O_2_ was able to promote hydroxylation reactions or to prevent extensive dehydroxylation and demethoxylation.

Analysis of the total compound class intensities for each investigated electrolyte system also allowed a more refined calculation of the rate of electrochemical degradation. The lignin degradation efficiency of TMS-2.5-H_2_O increased to 31% (w/w) using this approach as compared to simply using the yield of fraction 3 (see above). The degradation rate of [emim][OTf]-2.5-H_2_O, on the other hand, decreased to 19% (w/w) as calculated by the more refined analysis. Previously^40^, the composition of measured *m/z* features was restricted to C, H and O-containing molecules. Products from secondary reactions were excluded in this assignment and therefore skewed the calculated efficiency. LC-MS analysis of a raw mixture showed that total intensity (Fig. 4A) and product distribution (Figure S5) were comparable to the purified fractions. The lower total intensity of the raw mixture resulted from samples of lower concentrations, because at least 15% (w/w) of the lignin dissolved in ionic liquid were insoluble in the chromatographic solvents. Nevertheless, overall monitoring of the electrochemical decomposition process, without prior liquid-liquid extraction, was readily possible. TMS-0-H_2_O_2_ and TMS-2.5-H_2_O_2_ showed significant decreases of measured intensities and therefore effectiveness (TMS-0-H_2_O_2_, decrease of 13%; TMS-2.5-H_2_O_2_, decrease of 51%). These reductions most likely resulted from overexpression of the H_2_O_2_ oxidation. Low molecular weight mono- or dicarboxylic acids are main products of extensive H_2_O_2_ oxidations^64^. However, these low molecular weight carboxylic acids were only poorly detectable by the used analysis method, requiring a more suitable method in the future.

**Figure 5:**
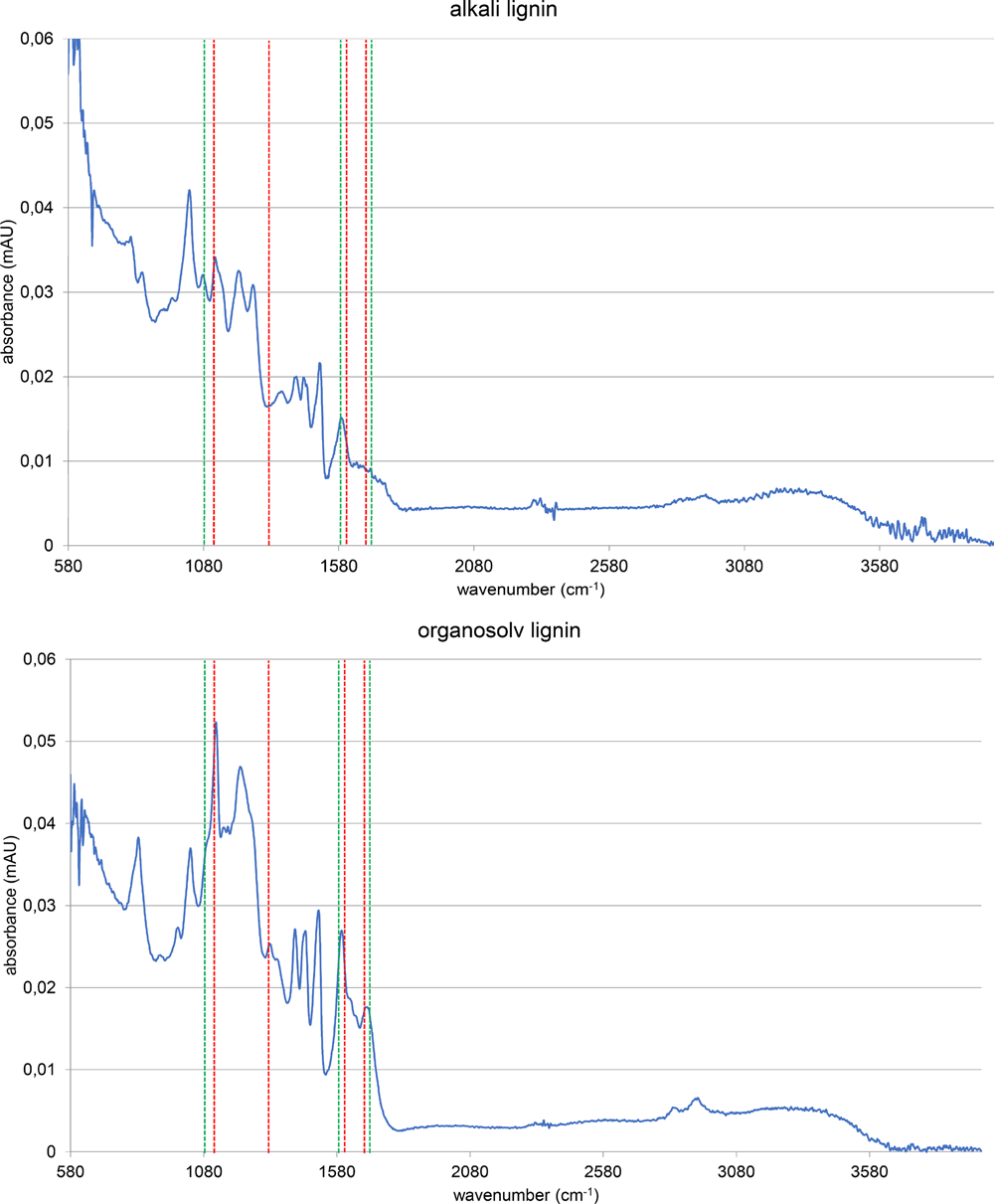
FTIR spectra of precursor alkali lignin (top) and Organosolv lignin (bottom). (Scan range: 500–4000 cm^−1^). The indicated absorbance bands were adapted from the literature^79^ (characteristic softwood absorbance bands are highlighted in green; characteristic hardwood absorbance bands are highlighted in red color).

Using different types of lignin provided important information on the general applicability of the implemented degradation processes. Degradation experiments of Organosolv lignin exhibited similar changes of chemical class distributions for each fraction and relative abundances (for chromatograms and mass spectra, see Figure S6; for total intensities and class distributions, refer to Figure S7). Both lignins exhibited FTIR absorbance bands characteristic for softwood and hardwood lignin (Fig. 5). By comparing the resulting FTIR spectra with the literature^79^, the alkali lignin was classified as softwood lignin and the Organosolv lignin as hardwood lignin. In addition to significant chemical class differences, the lignins also exhibited strong natural variations of structural compositions. Therefore, the spectrum of resulting degradation products (and the fraction of compounds, which were amenable to ionization) varied strongly between the samples. Nevertheless, the similarities with respect to observed changes of compound class distributions and relative intensities showed that the electrochemical approach was compatible with different lignins.

### Distribution of volatile, low molecular weight products

Detection of the electrochemically generated mono-aromatic molecules and dimers of low molecular weight was more difficult using LC-APCI-MS, because of the insufficient gas phase proton affinities that these molecules exhibit. Therefore, GC-MS was chosen as complementary analytical platform for detection of volatile, less polar molecules. The GC-MS results for fraction 3 are summarized in Table 3. The most obvious difference between the electrochemically-treated fraction 3 and the control was seen in the relative abundances of vanillin. While the control was dominated by vanillin (63% of total), there were additional abundant components after electrochemical decomposition mainly oxidized analogs of vanillin (homovanillic and vanillic acid). Such aldehyde oxidations to carboxylic acids are well known^80^. Degradation products containing *p*-hydroxyphenyl units primary resulted from electrochemical reduction of guaiacyl and syringyl core units, which were mainly seen in fraction 3 of TMS-2.5-H_2_O. These results were consistent with the dehydroxylation and demethoxylation reactions mentioned above. The electrophilic substitution of OH^+^, as described in Fig. 1, was confirmed by GC-MS analysis *via* detection of 1,2-benzendiol ([emim][OTf]-2.5-H_2_O, TMS-2.5-H_2_O) and 3,4-dihydroxybenzaldehyde (TMS-2.5-H_2_O) at significant levels. TMS-0-H_2_O_2_ and TMS-2.5-H_2_O_2_ experiments mainly yielded carboxylic acids, which was consistent with the literature^64^. Excessive H_2_O_2_ oxidation cracked the aromatic rings and formed these carboxylic acids.

**Table 3:** Compound distribution within fraction 3 for each electrolyte system using GC-MS. Main components (relative abundance >10%) are highlighted (bold-face).

compound	UT		[emim][OTF]-2.5-H_2_O		TMS-2.5-H_2_O		TMS-0-H_2_O_2_		TMS-2.5-H_2_O_2_	
	*rt* (min)	relative abundance (%)	*rt* (min)	relative abundance (%)	*rt* (min)	relative abundance (%)	*rt* (min)	relative abundance (%)	*rt* (min)	relative abundance (%)
2,2-dimethoxypropane	—	—	—	—	—	—	—	—	3.52	0.13
guaiacol	4.56	0.13	4.57	0.82	—	—	4.55	0.07	—	—
2-ethyl-2-methyl-1,3-cyclopentandione	—	—	5.31	0.24	—	—	—	—	—	—
4-ethylguaiacol	7.08	0.07	7.09	0.33	—	—	—	—	—	—
2-methoxy-4-vinylphenol	7.68	0.4	7.69	1.62	7.69	1.83	7.67	1.16	7.64	0.8
2-methoxy-4-allylphenol	8.16	0.11	—	—	—	—	—	—	—	—
syringol	8.39	0.11	—	—	—	—	—	—	—	—
1,2-benzenediol	—	—	8.56	3.69	8.75	4.47	—	—	—	—
**vanillin**	**9.45**	**63.21**	**9.39**	**30.37**	**9.31**	**21.57**	9.42	2.78	9.37	1.00
isoeugenol	—	—	—	—	—	—	10.04	0.27	10.05	0.11
4-propylguaiacol	10.17	0.17	10.18	0.37	10.17	0.48	10.19	0.50	10.19	0.31
**acetovanillone**	10.35	7.75	**10.37**	**12.03**	10.32	1.88	10.34	0.91	—	—
4-hydroxybenzaldehyde	10.66	0.64	—		10.64	1.58	—	—	—	—
homovanillyl alcohol	—	—	11.04	1.52	11.05	2.11	—	—	—	—
4-ethoxy-3-anisaldehyde	11.28	0.83	11.31	1.27	11.27	0.65	—	—	—	—
2,4-dihydroxy-3′-methoxyacetophenone	11.5	0.87	11.53	1.03	—	—	—	—	—	—
7-hydroxy-6-methoxy-1-benzofuran-3(2 H)-one	—	—	—	—	—	—	11.57	1.06	—	—
2-methoxyhydroquinone	—	—	—	—	11.61	1.93	—	—	—	—
dibenzyl ether	11.71	0.8	—	—	—	—	—	—	—	—
2,6-dimethoxy-4-(2-propenyl)-phenol	—	—	—	—	12.47	0.37	—	—	—	—
**homovanillic acid**	**12.72**	**15.77**	**12.76**	**34.68**	**12.69**	**25.24**	—	—	—	—
**vanillic acid**	—	—	13.13	6.09	**13.13**	**14.52**	13.11	0.67	13.11	1.49
3-methoxycinnamic acid	13.61	0.57	—	—	—	—	—	—	—	—
2,4-dimethoxyphenol	—	—	—	—	14.49	5.61	—	—	—	—
n-undecanoic acid	—	—	—	—	—	—	14.77	3.54	14.76	4.46
**3,4-dihydroxybenzaldehyde**	—	—	—	—	**15.41**	**11.8**	—	—	—	—
4a-methyl-1,2,3,4,4a,9,10,10a-octahydro-1-phenanthrenol	15.8	0.44	15.83	0.71	15.81	1.31	—	—	—	—
**2-hydroxycyclopenta-decanone**	—	—	—	—	—	—	**16.48**	**17.08**	**16.46**	**14.11**
6-ethoxy-1,4-dimethoxynaphthalene	—	—	16.62	1.23	—	—	—	—	—	—
tetradecanoic acid	—	—	—	—	—	—	16.67	2.45	16.66	3.22
4-hydroxymandelic acid	—	—	—	—	17.15	2	—	—	—	—
2-hydroxy-3-(3-methyl-2-butenyl)-3-cyclopenten-1-one	—	—	—	—	17.68	1.75	—	—	—	—
a-ethyl-p-methoxybenzyl alcohol	—	—	—		18.03	0.92	—	—	—	—
4-hydroxy-3-methoxyphenylacetylformic acid	—	—	18.1	0.01	—	—	—	—	—	—
abietic Acid	18.51	0.85	—	—	—	—	—	—	—	—
2,2′methylenebis[5-methyl-6-(2-methyl-2-propanyl)phenol]	—	—	—	—	—	—	—	—	18.89	2.49
ethyl homovanillate	—	—	—	—	18.99	5.48	—	—	—	—
palustric Acid	19.08	1.24	—		—	—	—	—	—	—
4′-methoxy-2-hydroxystilbene	—	—	19.08	1.17	—	—	19.05	7.62	—	—
**13-isopropylpodocarpa-8,11,13-trien-15-oic acid**	19.45	4.98	19.43	1.62	19.45	4.75	**19.44**	**37.92**	**19.43**	**59.95**
neopregnenolone	20.27	1.11	20.28	1.08	20.22	0.57	—	—	—	—
secoisolariciresinol	—	—	—	—	—	—	20.52	8.95	—	—

## Conclusions

In this study, electrochemical degradation of lignin using ionic liquid electrolyte systems was successfully performed. The lignins used were not representative for real biomass lignin, but were able to provide a proof of concept. The large spectrum of observed degradation reactions included several significant transformations that required the presence of water. Using a protic ionic liquid significantly reduced the number of reaction channels, since the presence of H^+^ transport reactions favored the hydrogen peroxide reaction pathway, while the protic IL remained otherwise unchanged and did not significantly undergo chemical reactions with the dissolved lignin. “Water-contaminated”, protic IL systems in combination with the improved solubility of lignin in the IL provided a platform for enhanced electrochemical depolymerization and offered great potential for (semi-)automated application. The exogenous addition of large amounts of H_2_O_2_, however, revealed that the electrochemical process can be significantly influenced, triggering significant consumption of the protic IL during degradation. Further studies will focus on the optimization of the system’s effectiveness by investigating different electrode materials, cell voltages and counter-anions. As well, the requirements of certain chemical functionalities for successful electrochemical reactions will be investigated by performing mechanistic studies on lignin-model compounds. In addition, real biomass lignins from the pulping industry will be investigated and electrochemically treated to confirm the broad applicability.

## Materials and Methods

### Materials

Methanol (HPLC grade) and aqueous hydrogen peroxide solution (technical, 33% w/w) were purchased from VWR (Darmstadt, Germany), acetonitrile (HPLC grade), triethylamine (>99%), titanium(IV) oxysulfate–sulfuric acid solution (27–31% H_2_SO_4_; ~5% based on Ti) and DMSO-d_6_ (99.9% D) from Sigma-Aldrich (Steinheim, Germany), methanesulfonic acid (70% aq.) from Carl Roth (Karlsruhe, Germany) magnesium sulfate (puriss, anhydrous) from an in-house university supply and diethyl ether (>99%) from Overlack (Mönchengladbach, Germany). Ultra-pure water was generated using an Elga (Celle, Germany) Purelab Ultra purification system. Acetanilid from Merck (Darmstadt, Germany) was used as calibration standard for elemental analysis. The aprotic ionic liquid 1-ethyl-3-methylimidazolium trifluoromethanesulfonate (99%) was purchased from Iolitec (Heilbronn, Germany) and was dried under vacuum conditions at 50 °C prior to the degradation process. The protic ionic liquid triethylammonium methanesulfonate was synthesized by dropwise neutralization of 1.1 equivalents of aqueous triethylamine solution with 1.0 equivalent of aqueous methanesulfonic acid at 0 °C. The resulting solution was stirred for 3 h. Excess of triethylamine and solvent were removed by rotary evaporation and the resulting residue was dried under vacuum at 70 °C for 2 d yielding the protic ionic liquid in quantitative amounts as a colorless crystalline solid. ^1^H-NMR (400 MHz, DMSO-d_6_): δ 9.30 (s, 1 H, NH), 3.41–2.95 (m, 6 H, CH_2_), 1.30 (t, J = 7.3 Hz, 9 H, -CH_3_); ^13^C-NMR (101 MHz, DMSO-d_6_) δ 45.71 (CH_2_), 38.89(SO_3_CH_3_), 8.55 (CH_2_ *C*H_3_). Reticulated vitreous carbon foam (thickness, 2.5 mm; porosity 96.5%; 24 pores/cm) was purchased from Goodfellow (Bad Nauheim, Germany). Alkali lignin (Sigma-Aldrich) and organosolv lignin (Chemicalpoint, (Deisenhofen, Germany) were dried under vacuum conditions for 3 d. Triethylamine was distilled prior to use. All other chemicals were used without prior purification.

### Electrochemical depolymerization

For electrochemical degradation, 1 g of lignin was dissolved in 10 g purified IL at 50 °C within 2 d. Two parallel reticulated vitreous carbon electrodes (46 × 14 mm) were placed into the investigated homogeneous solutions (no visible sedimentation within 2 w period). In all experiments, a constant distance of 5 mm was maintained between the two electrodes. To reduce the blend viscosity and to facilitate the stirring process, the solutions were heated to 65 °C and spiked with 1 mL of water, to obtain “water contaminated” electrolyte system. The spiked solutions were stirred until full homogeneity was achieved and the resulting liquid level was marked. The electrochemical reactions were performed at a cell voltage of 2.5 V on a VMP3 potentiostat (BioLogic Science Instruments, Seyssinet-Pariset, France) for 1 h under intensive magnetic stirring using a MR3001 K stirring device from Heidolph (Schwabach, Germany). After 1 h, the liquid level was refilled with water to the initial level and the system was stirred until a homogeneous solution was obtained. This cycle from electrochemical treatment to water addition was repeated for 24 times. After 24 h, the treated solutions were mixed with 250 mL of methanol, vigorously stirred for 1 h and subsequently filtered using filter paper MN617 ¼ from Macherey-Nagel (Düren, Germany). The solid residues (insoluble fractions) were dried under ambient conditions and the solvent of the filtrate was removed by rotary evaporation. The resulting homogeneous lignin/ionic liquid solution was added to 250 mL of deionized water, stirred for 1 h and filtered. The aqueous phase was extracted with 200 mL of diethyl ether, the organic phase separated and dried with magnesium sulfate. After air drying, the solid residue was treated with 200 mL of diethyl ether for 16 h to extract the organic compounds of lower molecular weights. After filtration, the solid residues were dried (methanol fraction). The organic solvent of the filtrate was dried with magnesium sulfate, filtered and combined with the diethyl extracts of the aqueous phase. The solvent of the combined diethyl ether phases was removed by rotary evaporation, resulting in a brownish solid (Et_2_O fraction).

### Cyclic voltammetry

CV experiments were performed on a VMP3 potentiostat (BioLogic Science Instruments, Seyssinet-Pariset, France) and a three-electrode electrochemical cell. The reference electrode was Ag/AgCl (KCl 3.0 M). A platinum wire was used as an auxiliary electrode and the working electrode was a reticulated vitreous carbon electrode (46 × 14 mm). The anhydrous ILs triethylammonium methanesulfonate and 1-ethyl-3-methylimidazolium trifluoromethanesulfonate were thoroughly deoxygenated with pure nitrogen for about 30 min prior to each experiment.

### Photometric H_2_O_2_ determination

H_2_O_2_ calibration standards (0.1, 0.25, 0.5, 1.0, 2.5 μmol/L) were prepared by diluting aqueous hydrogen peroxide stock solutions (0.1 mol/L) with TMS. A pure TMS solution was used as a blank for photometric analysis. Fifty μL of acidic titanium(IV) oxysulfate solution (~5% based on Ti) were added to freshly prepared solutions and the resulting yellow solution measured on a Varian (Waldbronn, Germany) Cary 50 Scan UV-Vis spectrometer at *λ* = 415 nm. For determining the electrochemically-generated H_2_O_2_, 1 mL of water was added to 10 mL of TMS, flowed by electrochemical treatment for 1 h. Analogous to the calibration solutions, 50 μL of acidic titanium(IV) oxysulfate solution (~5%) were added to the solution and was measured at *λ* = 415 nm.

### Elemental analysis, FTIR analysis and analysis of volatile content *via* GC-MS

Carbon, hydrogen, and nitrogen (CHN) content of the solid materials after liquid-liquid extractions were determined by elemental analysis using a Vario EL analyzer from Elementar (Haunau, Germany). FTIR analyses of the powdered samples were conducted on a Perkin Elmer (Walluf, Germany) Frontier spectrometer with ATR accessory in the mid IR range. The samples were slightly pressed and placed on a barium fluoride single crystal. Forty scans with resolution of 0.8 cm^−1^ were collected and co-added. For GC-MS, 1 mg of the respective fraction was dissolved in 1 mL of methanol and the precipitated residue was filtrated. One μL of the resulting solutions was injected onto a Agilent (Waldbronn, Germany) HP-5MS column (25 m × 0.320 mm i.d.; film thickness, 0.52 μm) and separation performed using a Thermo Fisher Scientific (Dreieich, Germany) Focus GC equipped with autosampler AI3000 and DSQ II mass spectrometer. The analyses were performed using a split ratio of 1:10 with helium at 1 mL/min. The inlet temperature was kept at 280 °C, MS transfer line at 250 °C, and ion source at 200 °C. The temperature gradient started at 70 °C and was held there for 1 min, then raised to 260 °C at 10 °C/min, and held there for a further 1 min. The MS was operated in EI mode (70 eV, *m/z* 40–500) and scanned at 2022 u/s. Data acquisition started at 2.5 min after injection. Peak identification was performed using the NIST database.

### Analysis of small molecules (<1000 Da) *via* LC-HRMS

The solid precipitates (100 μg) after diethylether extraction and methanol-water precipitation were dissolved in 1 mL of methanol. Ten μL of this solution were injected onto a custom-made trioctylpropylphosphonium trifluoromethanesulfonate (SilPrPhoOTf) column and separated using gradient elution on an Agilent 1100 HPLC system, equipped with binary pump, degasser, autosampler, and variable wavelength detector. The column was heated to 40 °C using a Knauer (Berlin, Germany) thermostat. The HPLC was connected to a solariX 7 Tesla FTICR instrument from Bruker (Bremen, Germany), equipped with APCI source and Infinity cell. The gradient started at 10% B for 10 min, increased to 30% B within 5 min, held there for 5 min, increased to 80% B within 25 min, held there for 25 min, and then reduced to 10% B within 0.1 min followed by equilibration period for 25 min at 10% B. LC-MS experiments were performed in negative ionization mode using a flow rate of 0.25 mL/min. For each spectrum, 2 transients were collected and co-added, giving an estimated resolving power of 140,000 at *m/z* 400. Mass spectra were externally calibrated using the Agilent APCI/APPI tuning mix solution. Elemental restrictions for chemical compositions and data analyses using Bruker’s Data Analysis 4.2 were adopted from a previous study^40^.

## Electronic supplementary material

Supplementary Information
