# Supplementary material for: Sustainable Electrochemical Depolymerization of Lignin in Reusable Ionic Liquids
Source: Sci Rep. 2017 Jul 11;7:5041. doi: 10.1038/s41598-017-05316-x (PMC5505966; doi:10.1038/s41598-017-05316-x)
Supplement: Supplementary file 1 — Supplementary Information [file 41598_2017_5316_MOESM1_ESM.pdf]

# *Electronic Supplementary Information*

# Sustainable Electrochemical Depolymerization of Lignin in Reusable Ionic Liquids

**Tobias K.F. Dier,[1] Daniel Rauber,[2] Dan Durneata,[2] Rolf Hempelmann,[2] Dietrich A. Volmer[1]**

[1]Institute of Bioanalytical Chemistry, Saarland University, Campus B2.2, 66123 Saarbrücken, Germany. [2]Institute of Physical Chemistry, Saarland University, Campus B2.2, 66123 Saarbrücken, Germany. Correspondence and requests for materials should be addressed to D.A.V. (email: Dietrich.Volmer@mx.uni-saarland.de)

**Figure S1**. Calibration curve of the titanium(IV)-hydrogen peroxide complex using UV/Vis spectrometry at 415 nm........................................................................................................2

**Figure S2**. NMR spectra of 1-ethyl-3-methylimidazolium trifluoromethanesulfonate ([emim][OTf]) after and prior to electrochemical treatment……………………………….........3

**Figure S3**. NMR spectra of triethylammonium methanesulfonate (TMS) after and prior to electrochemical treatment .........................................................................................4

**Figure S4**. Base peak chromatograms and averaged mass spectra for each degradation process. ..........................................................................................................................5

**Figure S5**. Overlay of Kendrick mass defect data of the purified fraction 3 from TMS-2.5-$H_2O$ and the rcorresponding raw mixture after electrochemical treatment using 2D mass defect filteringt. ..........................................................................................................................6

**Figure S6**. Base peak chromatograms and averaged mass spectra for each degradation process.. .........................................................................................................................7

**Figure S7. A** Total intensity for relevant lignin degradation products. **B** Relative distributions (%) of chemical classes for fraction 2. **C** Relative distributions (%) of classes for fraction 3.. ........................8

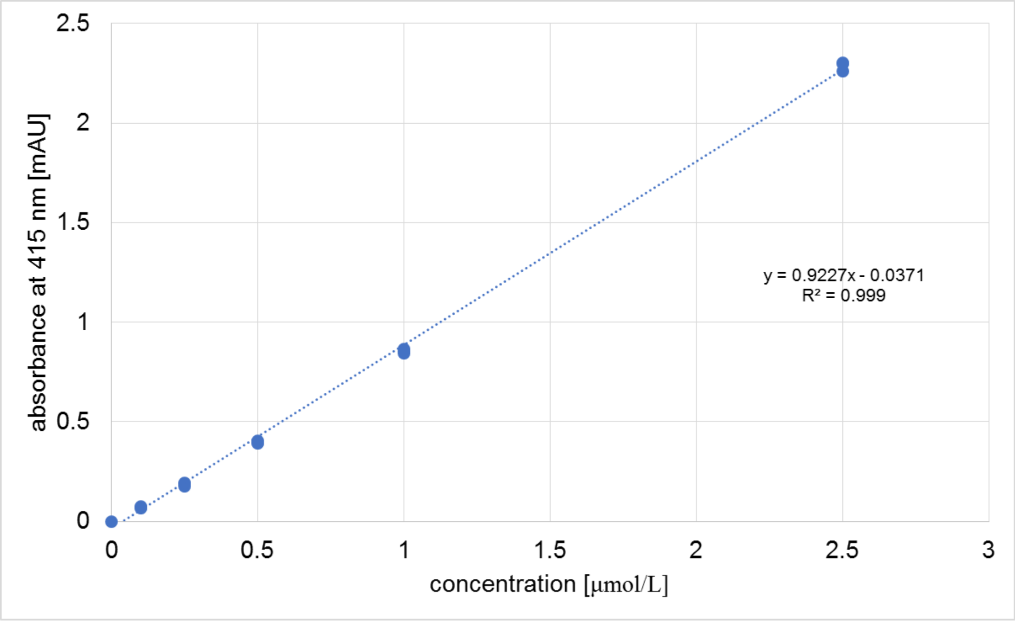


**Figure 1.** Calibration curve of the titanium(IV)-hydrogen peroxide complex using UV/Vis spectrometry at 415 nm. Concentrations of the calibration solutions: 0.0, 0.1, 0.25, 0.5, 1.0, and 2.5 µmol/L.

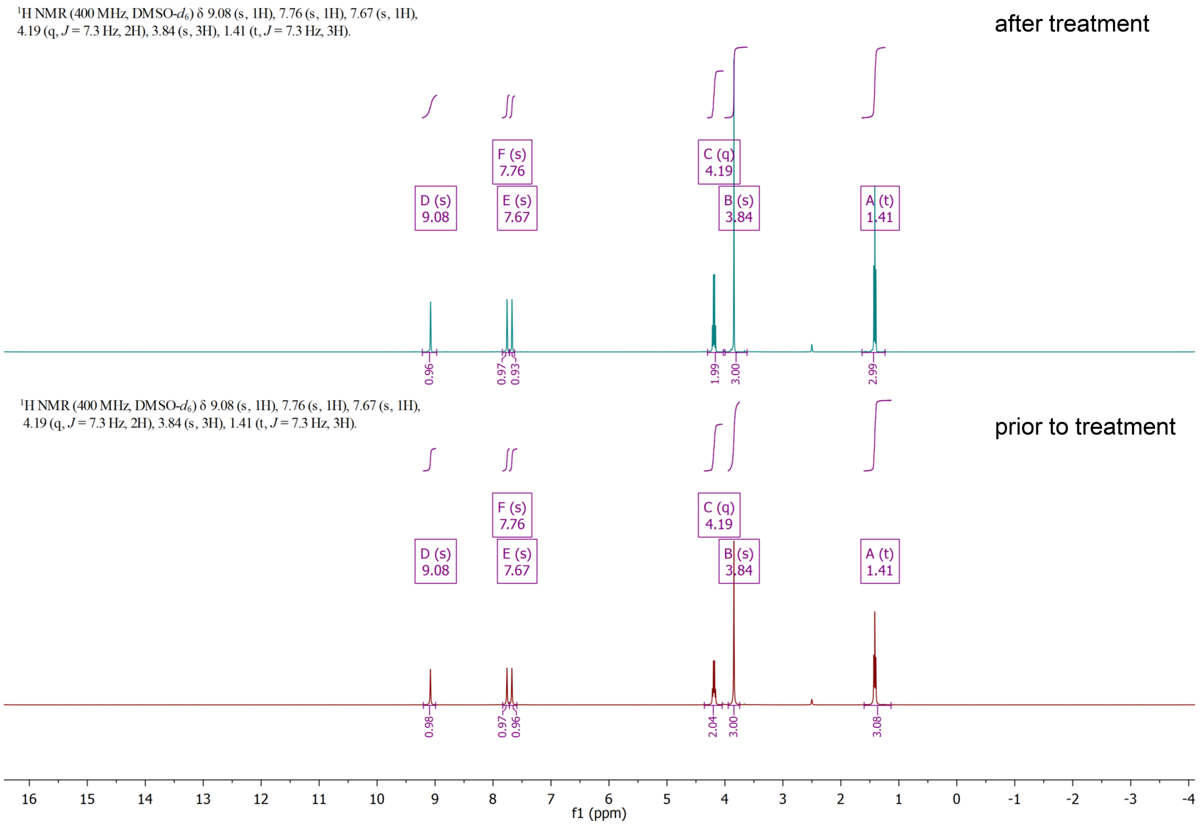


**Figure S2.** NMR spectra of 1-ethyl-3-methylimidazolium trifluoromethanesulfonate ([emim][OTf]) after (top) and prior to electrochemical treatment (bottom).

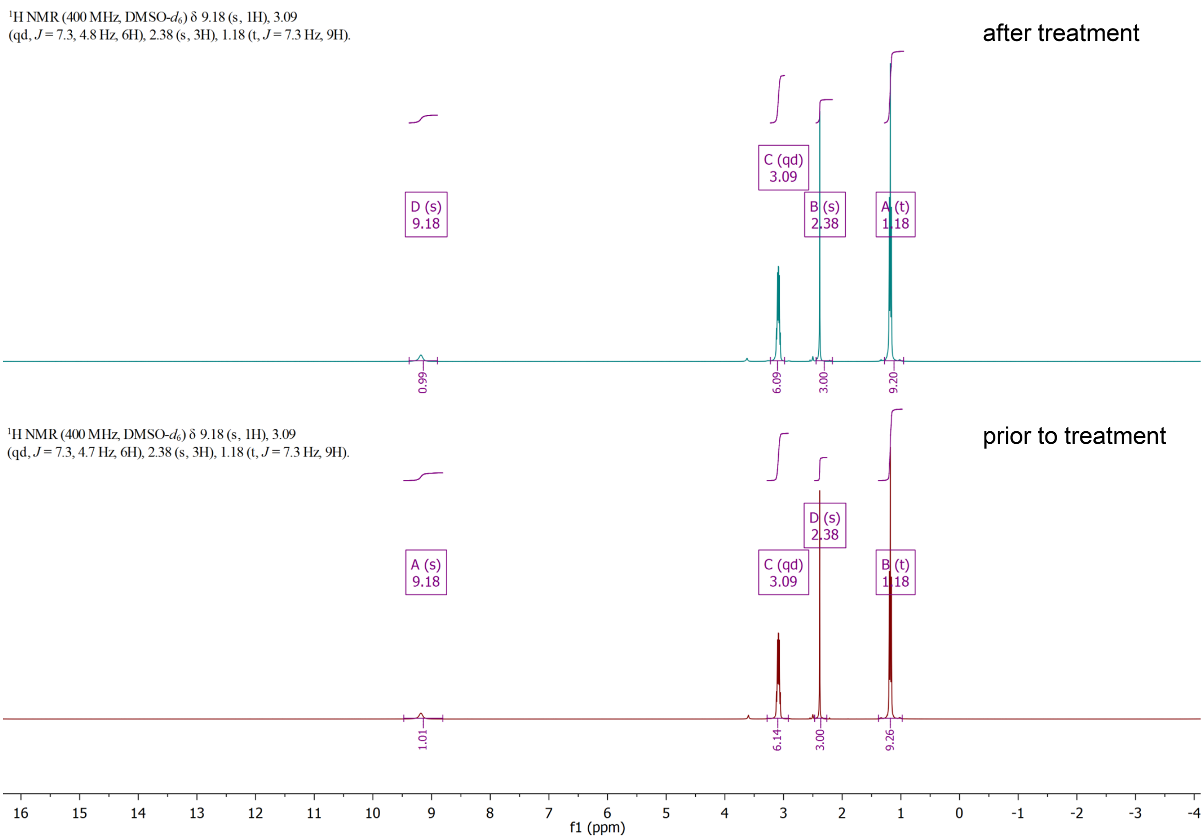


**Figure S3.** NMR spectra of triethylammonium methanesulfonate (TMS) after (top) and prior to electrochemical treatment (bottom).

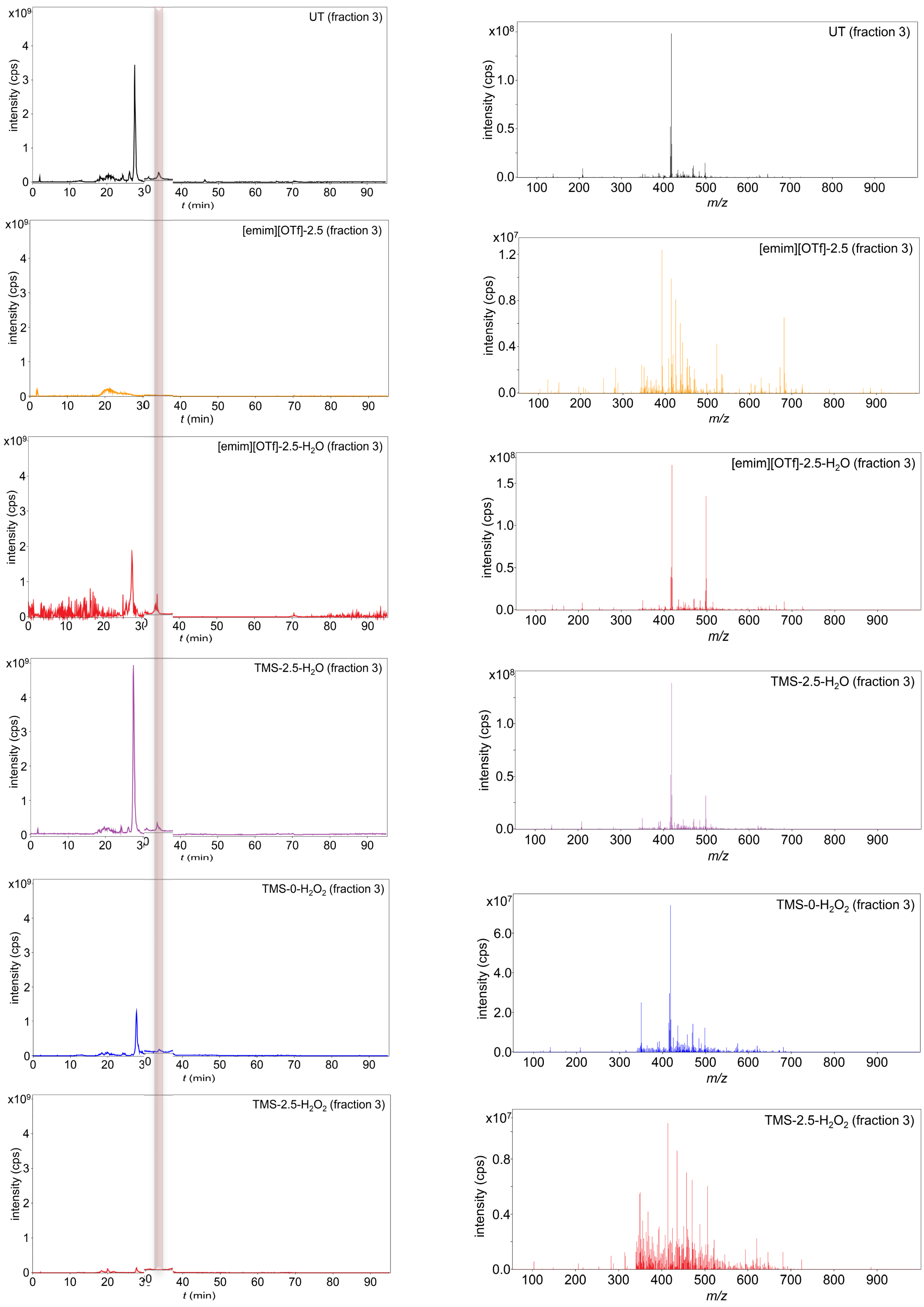


**Figure S4.** Base peak chromatograms (left) and averaged mass spectra (retention time, 34 min) (right) for each performed degradation process. Alkali lignin was used for all experiments.

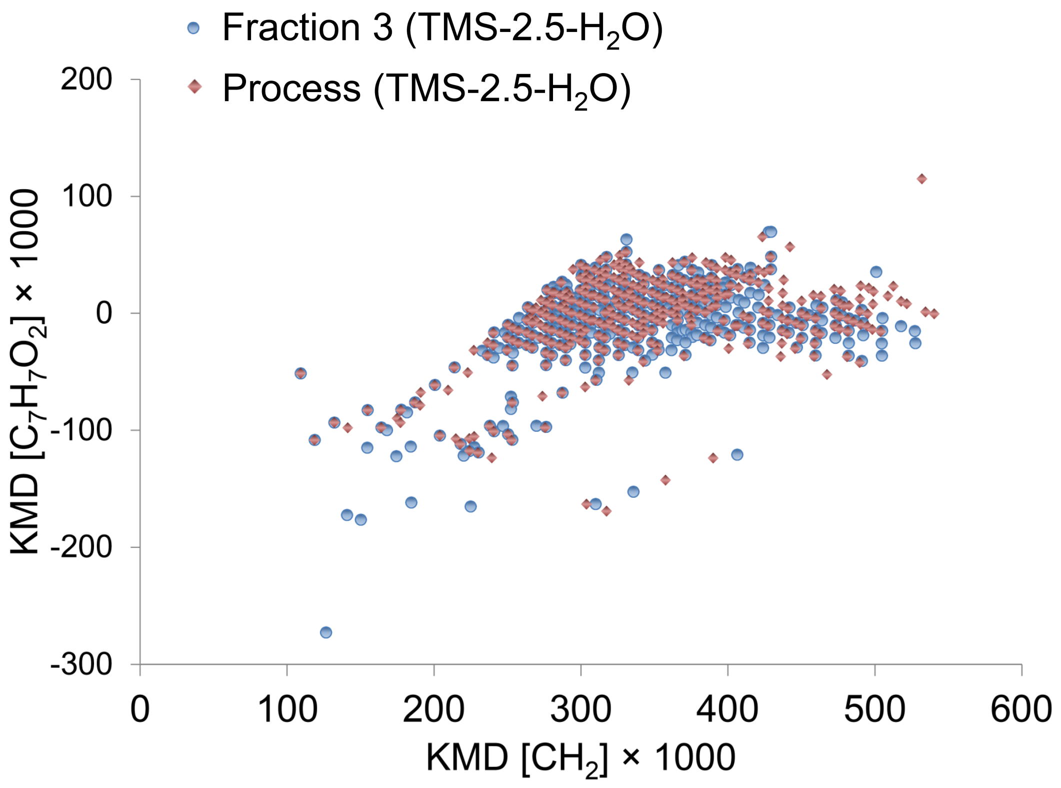


**Figure S5.** Overlay of Kendrick mass defect data of the purified fraction 3 from TMS-2.5-$H_2O$ and the corresponding raw mixture after electrochemical treatment using 2D mass defect filtering.

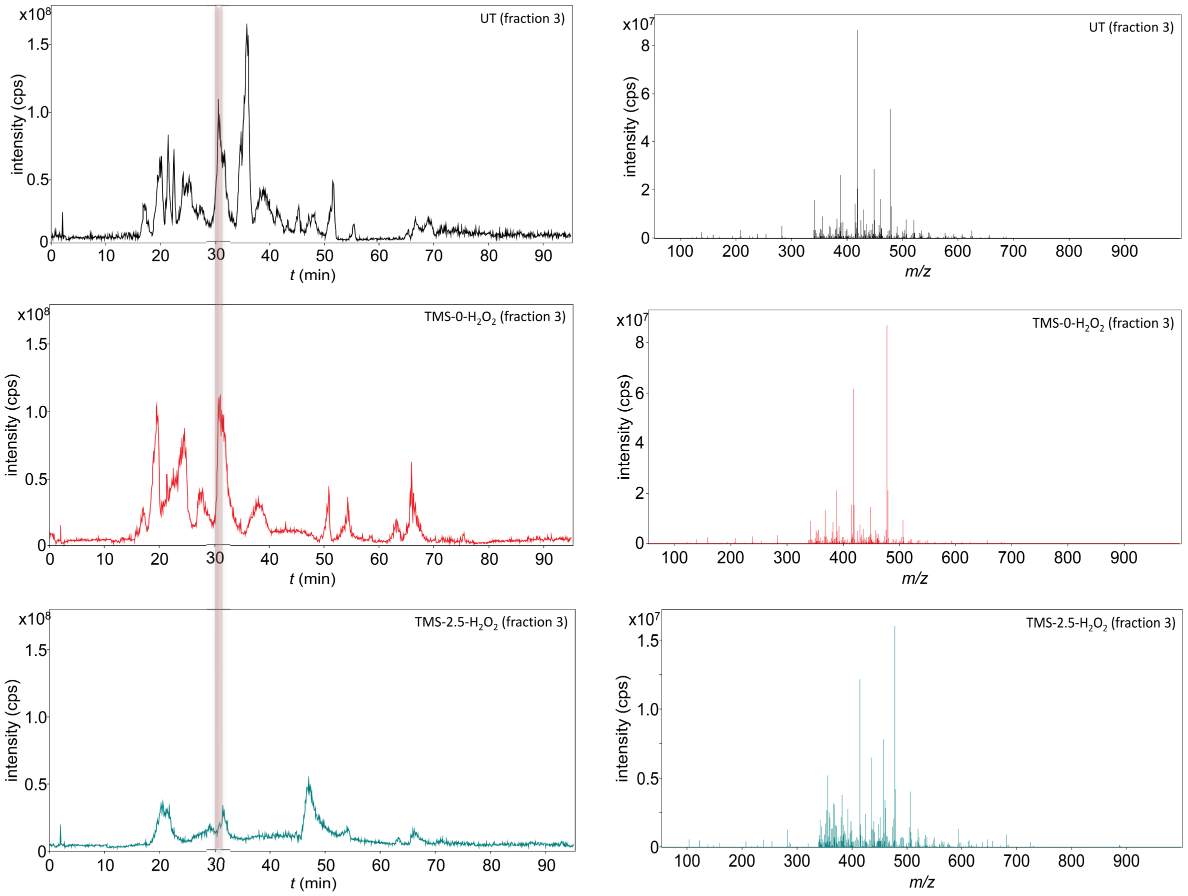


**Figure S6.** Base peak chromatograms (left) and averaged mass spectra (retention time, 30.5 min) (right) for each degradation process. Organosolv lignin was used for all experiments.

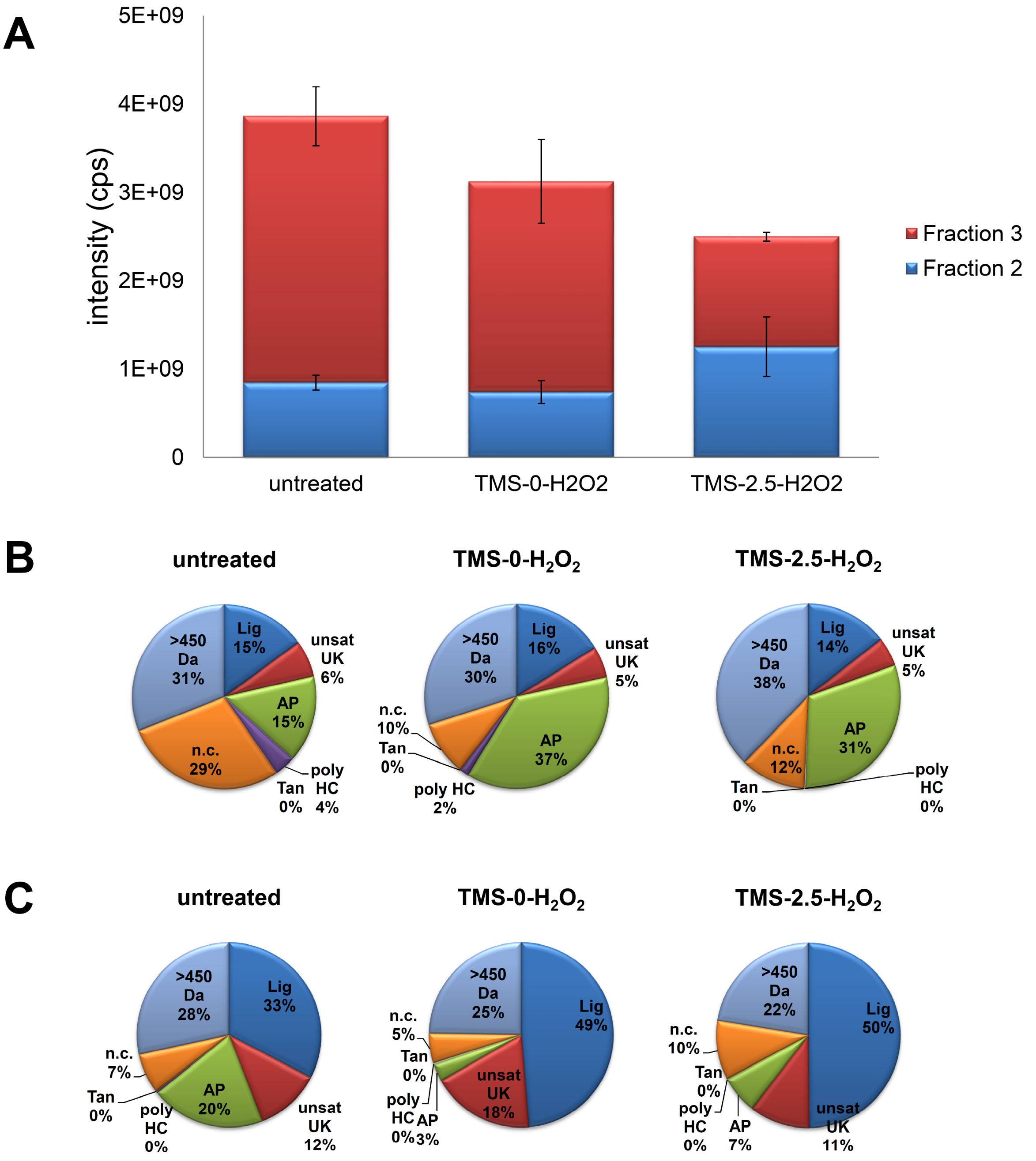


**Figure S7. A** Total intensity for relevant lignin degradation products (mass concentration, $\beta$=100 µg/mL). **B** Relative distributions (%) of chemical classes for fraction 2. **C** Relative distributions (%) of chemical classes for fraction 3. Distributions were restricted to $m/z \leq 450$, unless otherwise specified. Organosolv lignin was used for all experiments.
